# Aligning community-engaged research competencies with online training resources across the Clinical and Translational Science Award Consortium

**DOI:** 10.1017/cts.2020.538

**Published:** 2020-09-11

**Authors:** Rebecca J. Piasecki, Elisa D. Quarles, Mona N. Bahouth, Anwesha Nandi, Alicia Bilheimer, Lori Carter-Edwards, Cheryl R. Dennison-Himmelfarb

**Affiliations:** 1 School of Nursing, Johns Hopkins University, Baltimore, MD, USA; 2 Institute for Clinical and Translational Research, Johns Hopkins University, Baltimore, MD, USA; 3 North Carolina Translational and Clinical Sciences Institute, University of North Carolina at Chapel Hill, Chapel Hill, NC, USA; 4 School of Medicine, Johns Hopkins University, Baltimore, MD, USA

**Keywords:** Community-engaged research, community engagement, stakeholder engagement, training, education

## Abstract

**Introduction:**

The extent to which Clinical and Translational Science Award (CTSA) programs offer publicly accessible online resources for training in community-engaged research (CEnR) core competencies is unknown. This study cataloged publicly accessible online CEnR resources from CTSAs and mapped resources to CEnR core competency domains.

**Methods:**

Following a search and review of the current literature regarding CEnR competencies, CEnR core competency domains were identified and defined. A systematic review of publicly accessible online CEnR resources from all 64 current CTSAs was conducted between July 2018 and May 2019. Resource content was independently reviewed by two reviewers and scored for the inclusion of each CEnR core competency domain. Domain scores across all resources were assessed using descriptive statistics.

**Results:**

Eight CEnR core competency domains were identified. Overall, 214 CEnR resources publicly accessible online from 35 CTSAs were eligible for review. Scoring discrepancies for at least one domain within a resource initially occurred in 51% of resources. “CEnR methods” (50.5%) and “Knowledge and relationships with communities” (40.2%) were the most frequently addressed domains, while “CEnR program evaluation” (12.1%) and “Dissemination and advocacy” (11.2%) were the least frequently addressed domains. Additionally, challenges were noted in navigating CTSA websites to access CEnR resources, and CEnR competency nomenclature was not standardized.

**Conclusions:**

Our findings guide CEnR stakeholders to identify publicly accessible online resources and gaps to address in CEnR resource development. Standardized nomenclature for CEnR competency is needed for effective CEnR resource classification. Uniform organization of CTSA websites may maximize navigability.

## Introduction

Community-engaged research (CEnR) involves community stakeholders and researchers collaborating to co-design, implement, evaluate, and disseminate clinical and translational research that is culturally appropriate, valued, efficient, and effective [1]. CEnR is an important avenue for both researchers and community stakeholders to generate and disseminate knowledge in order to improve the health and well-being of patients and communities [2, 3]. There has been increased interest among academic institutions and community stakeholders to conduct CEnR. Supported by the National Institutes of Health, the Clinical and Translational Science Award (CTSA) program currently funds 64 institutions across the USA to further the progress of translational research [4]. One of the primary goals of the CTSA program is to foster CEnR by engaging patients and communities in every phase of the translational science and research processes [5, 6].

Establishing successful community–academic partnerships in research requires training, skills, and resources. With community engagement as a core component of all CTSA institutions, much of the work done across CTSAs in CEnR involves the development, testing, and dissemination of trainings and resources to enhance CEnR skills and knowledge among researcher and stakeholder populations, with the end goal of ensuring more successful community–academic partnerships. For more communities and researchers to effectively collaborate in CEnR, increasing access to quality CEnR education and training is crucial [1]. Therefore, the availability of resources online that are readily accessible to researchers and communities and contain accurate, up-to-date content is an essential part of the continued success of CEnR [7]. To this point, there is a focus by the National Center for Advancing Translational Sciences (NCATS) on shared resources and information to help advance CEnR [8]. However, the extent to which existing CEnR resources are publicly accessible online through CTSAs, and how these resources address CEnR core competencies is unclear.

The purpose of this study was to understand the extent to which CTSA programs offer publicly accessible online resources for training in CEnR core competencies. In this study, the term CEnR was used to refer to all forms of related research (e.g., community-based participatory research) [1]. The study purpose was addressed by examining the following aims: (1) identify all CEnR resources currently offered by CTSAs, with a focus on publicly accessible online resources and (2) systematically review publicly accessible online CEnR resources for whether resource content addressed identified CEnR core competency domains.

## Materials and Methods

### Study Design

Literature specific to CEnR education and training competencies was used to identify CEnR core competency domains. In April 2018, a search of PubMed was performed using the following search terms: community-based participatory research, community engagement, and competency. We also searched references cited in selected articles. An article was included if the primary focus was discussing competencies for training and performing CEnR. A total of eight articles were included for the final review. Common competencies and key attributes discussed in these articles were synthesized to identify core domains based on the literature [2, 6, 8–13]. Definitions and key aspects of domains were then agreed upon by research team members. The final eight CEnR core competency domains included: knowledge and perceptions of CEnR; personal traits necessary for CEnR; knowledge and relationships with communities; training of those involved in CEnR; CEnR methods; CEnR program evaluation; resource sharing and communication; and dissemination and advocacy (Table 1).

**Table 1.** CEnR core competency domain definitions and characteristics

**Knowledge and perceptions of CEnR**
Basic principles and concepts integral to understanding and performing CEnR, including: Value of CEnR, History of CEnR, CEnR approaches, Variations on CEnR (i.e., CEnR).
**Personal traits necessary for CEnR**
Personal attributes essential to successful CEnR participation, including: Introspection and openness, Flexibility, Willingness to share power and collaborate, Honesty and transparency, Willingness to teach and mentor, Clear communication (relaying information, negotiating, and listening), Cultural competency, Cultural humility.
**Knowledge and relationships with communities**
Stakeholder relationships and their involvement in CEnR, including: Understanding community character, culture, preferences, and context, Understanding organizational culture and context, Selecting and preparing community partners and other stakeholders, Initiating and building academic–community relationships, Developing and working with community advisory boards, Involvement of community before and during all phases of research project, Sustaining community–academic partnerships.
**Training of those involved in CEnR**
Ability of academic–community partners to successfully incorporate new stakeholders in CEnR project and processes, including: Identification and evaluation of stakeholders’ baseline skills and experiences relevant CEnR, Definition of roles and expectations for stakeholders involved in project, Plans for training new stakeholders, measuring effectiveness of that stakeholder training, Identification of resources necessary and available for stakeholder training.
**CEnR methods**
Technical components and skills required to perform CEnR, including: Understanding CEnR theoretical frameworks, Understanding and using community assessments, Agenda selection for research with community partners, Study design with community partners, Understanding key principles of relevant research methods, Performing literature reviews, Obtaining IRB approval, Research ethics, Understanding key components of participant recruitment, consent, and retention, Data management, Data analysis, Managing budgets, personnel, and resources.
**CEnR program evaluation**
Evaluating research project impact with intended stakeholders, as well as evaluation of efficacy of community–academic partnerships.
**Resource sharing and communication**
Facilitating equitable sharing of funding, resources, and credit involved in CEnR projects, including: Elucidating stakeholder expectations, Delineating roles, Disseminating funding throughout grant writing, budget development, and award processes, Understanding stakeholders time and resources.
**Dissemination and advocacy**
Methods for effectively communicating CEnR results to leverage positive change for key stakeholders are the following: Use of scholarly and lay media to relay findings to academic, community, and political audiences, Use of networking and outreach activities to communicate CEnR findings, Report lasting impacts of CEnR to help inform evidence-based policy change in the interests of key stakeholders.

CEnR, community-engaged research.

We then conducted a systematic review of CEnR resources publicly accessible online between July 2018 and May 2019 from the websites of all 64 currently funded CTSAs to identify CEnR-specific resources. Resource characteristics were recorded including institution name, link to resource, ability to download resource, intended audience for resource, and target population of interest for a resource. Resources were included for review and analysis if all components of the resource were fully available online; they were primarily intended for education or training in performing CEnR; they were directly developed, co-developed, or sponsored by a CTSA; and they were directly available through a CTSA website. A CEnR resource was determined to be publicly accessible online if all components of the resource were available online. Resources were excluded if the website used to access the resource required the study team to request information or permission from the host CTSA in order to access the resource, and that access was not received within 5 days; the resource required in-person training, or blended online components with off-line formats, such as in-person training; or, the resource was not intended for CEnR education and training.

**Fig. 1.**
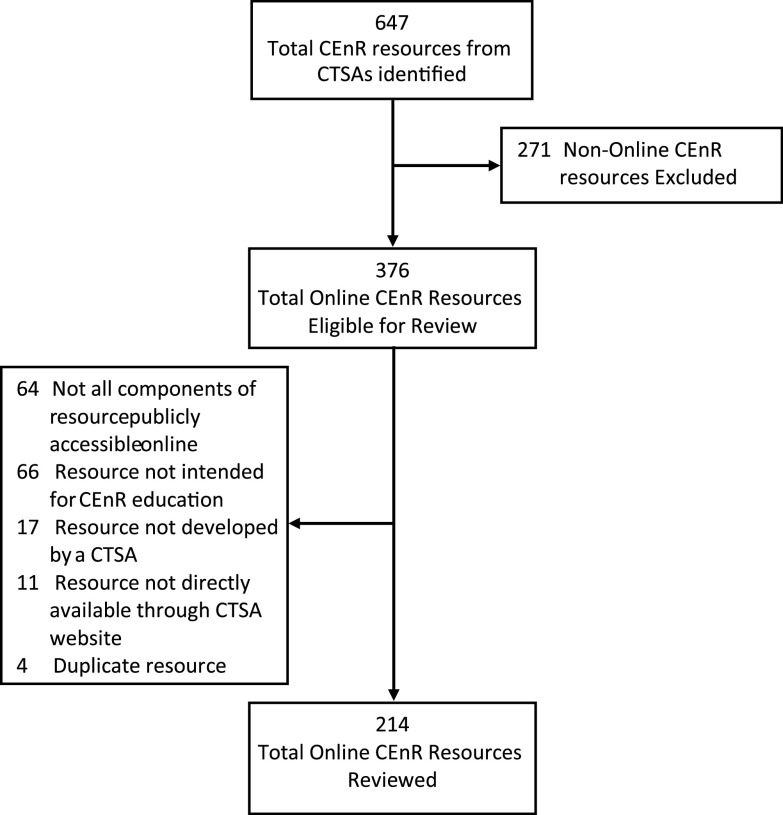
Flowchart for the inclusion of publicly accessible online resources for CEnR. CEnR, community-engaged research; CTSA, Clinical and Translational Science Award.

Overall, 647 CEnR resources available between July 2018 and May 2019 from 64 CTSAs were identified. Of those, 376 resources were identified as being publicly accessible online. A total of 214 of 647 (33.1%) publicly accessible online CEnR resources from 35 of 64 (54.7%) CTSAs met the criteria for inclusion in review (Fig. 1) [14-48]. Most resources reviewed were in the text (i.e., downloadable literature, PowerPoints, etc.), webinar, or recorded lecture format.

For each resource, the CEnR core competency domains addressed were evaluated. For each resource included in the final sample, the content was independently reviewed by two reviewers and subsequently scored by the extent to which it addressed each CEnR core competency domain using a dichotomous score as follows: (1) Addressed – the domain was the main topic discussed in the resource, or was substantively discussed, but was not the main focus of the resource or (2) Not addressed – minimally discussed (i.e., only discussed in a few sentences or bullet points) or not discussed at all. Reviewers’ domain scores for the same resource across reviewers were then compared. To resolve scoring discrepancies, reviewers of the resource convened to reach an agreement on the final domain score.

Finally, reviewers were asked to keep field notes regarding observations made in accessing and reviewing resources. These notes were then compared and discussed by the study team to determine what, if any, common themes were noted among reviewers.

IRB approval for this study was not required.

### Statistical Analysis

Domain scores for study sample resources were aggregated and analyzed with descriptive statistics (frequencies and percentages) to assess the extent to which each of the eight identified CEnR core competency domains was addressed across resources.

## Results

Of the 214 resources included for review, 60% (*n* = 130) were directly downloadable documents. The remaining 40% required additional steps to access the resource (i.e., a video played on YouTube or an interactive module that could not be downloaded) and the resource itself was “housed” online.

The intended audiences for the resources reviewed included community partners (33%; *n* = 71), academic partners (62%; *n* = 134), and clinician partners (8%; *n* = 18). For 13% (*n* = 29) of resources, the intended audience was unclear. About 12% (*n* = 27) of resources discussed the conduct of CEnR with specific communities or populations, including Latinx communities, African-Americans, and military veterans.

**Fig. 2.**
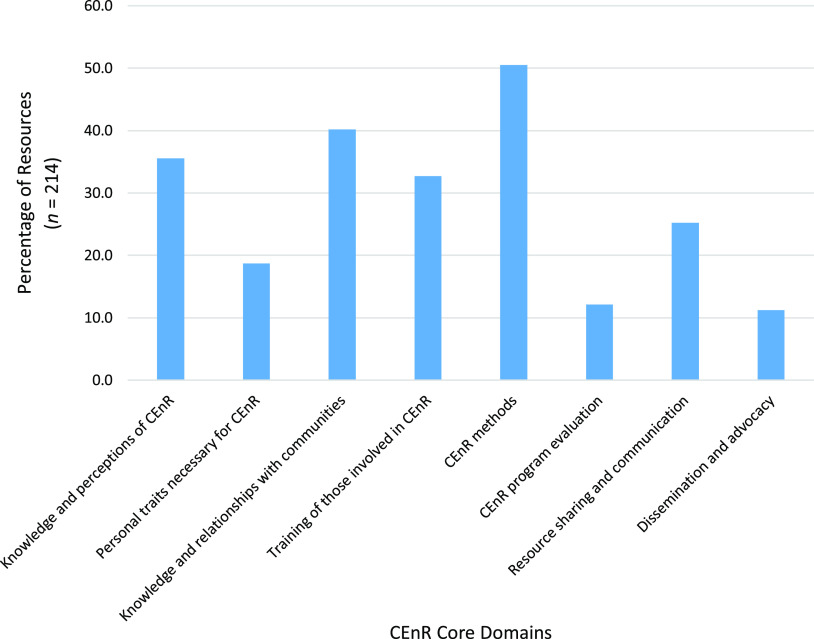
Percentage of core domains addressed across all sample resources by domain. CEnR, community-engaged research.

The CEnR core competency domains most commonly addressed in the study sample of resources were “CEnR methods” (50.5%; *n* = 108) and “Knowledge and relationships with communities” (40.2%; *n* = 86). The CEnR core competency domains least commonly addressed in the study sample of resources were “Dissemination and advocacy” (11.2%; *n* = 24) and “CEnR program evaluation” (12.1%; *n* = 26). Further details regarding the percentage of core domains addressed across all sample resources are provided in Fig. 2.

**Fig. 3.**
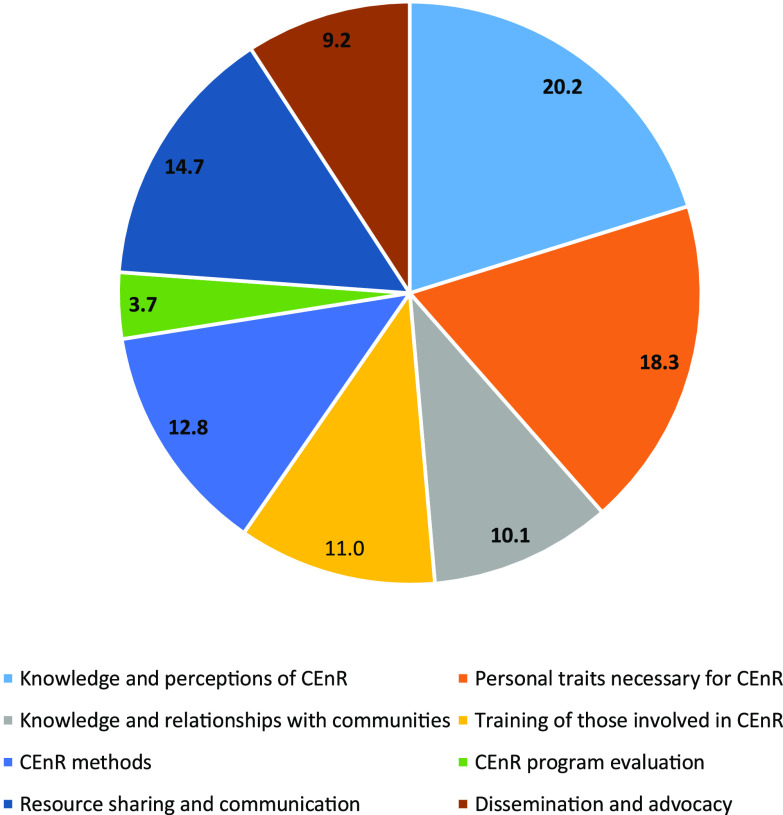
Initial reviewer discrepancies scoring the extent to which domains were addressed within resources by percentage. CEnR, community-engaged research.

Comparison of reviewers’ field notes revealed that a lack of standardized CEnR nomenclature became problematic when reviewing and scoring resources. Scoring discrepancies for at least one domain within a resource initially occurred in 50.9% (*n* = 109) of resources. All scoring discrepancies were resolved. Of the resources in which scoring discrepancies initially occurred, 71.6% (*n* = 78) were ultimately determined to address the domain in question, while 28.4% (*n* = 31) were ultimately determined to not address the domain in question. The core domains with the most observed initial scoring discrepancies included “Knowledge and perceptions of CEnR” (20.2%; *n* = 22) and “Personal traits necessary for CEnR” (18.3%; *n* = 20). The core domains with the fewest initial scoring discrepancies were “CEnR program evaluation” (3.7%; *n* = 4) and “Dissemination and advocacy” (9.2%; *n* = 10). Further details regarding initial reviewer discrepancies in scoring the extent to which domains were addressed within resources are provided in Fig. 3.

Additionally, the comparison of reviewers’ field notes revealed CTSA website navigability played a major role in excluding potential resources from the analysis. Issues related to the navigability of CTSA websites that were noted by reviewers included: inconsistent organization and nomenclature related to labeling CEnR resources, convoluted hyperlinks and website organization to access CEnR resources, and restricted access to CEnR resources (e.g., needed faculty/staff credentials to access resources).

## Discussion

Engagement of communities across the continuum of translational research is vital in ensuring that such research meaningfully improves the health and well-being of those communities [49]. To accomplish this, community and academic partners must understand all aspects of CEnR so that community partners are able to understand and actively contribute to all phases of the research process. Education and training in every domain of CEnR core competency are then essential for both communities and researchers to ensure that all stakeholders are competent and comfortable contributing to CEnR.

Through this study, we generated a comprehensive inventory of CEnR resources that are publicly accessible online to researchers and other stakeholders interested in learning about and conducting CEnR. Our comprehensive review of these resources elucidated the CEnR core competency domains that are addressed in currently available resources. Results suggest that CTSAs provide numerous resources specific to CEnR through online formats. “CEnR methods” and “Knowledge and relationships with communities” were the most commonly addressed domains by these online resources. Our findings also indicate that major gaps exist in resources addressing the domains of “CEnR program evaluation” and “Dissemination and advocacy.” Furthermore, the domains of “Knowledge and perceptions of CEnR” and “Personal traits necessary for CEnR” were difficult to initially score with consistency. Finally, considering that only 33.1% of CEnR resources reviewed were publicly accessible online, and those resources only came from 54.7% of CTSAs, this demonstrates that there is a need for CTSAs to substantially improve public access to online CEnR resources.

Several challenges pertaining to accessing and classifying publicly accessible online CEnR resources were noted. First, the original source of the materials was not always clear to the reviewer. While many tools were created by the institution hosting them on their website, some resources were shared from other sources and thus redundant between sites. In the future, it will be useful for tools to be identified with the date of creation and source institution(s) so stakeholders can identify content developers and content modifications to facilitate adaptation and dissemination of CEnR resources. Second, we encountered challenges in successfully navigating CTSA websites to access CEnR resources. Marked differences were observed in how CTSA websites were structured to house and allow access to CEnR resources which led to challenges in navigation. Third, several CTSA websites offered CEnR resources by providing “hyperlinks to hyperlinks,” where instead of the resource being directly available through the CTSA website, one would have to follow several hyperlinks in order to locate the original resource of interest. Even among our trained reviewers and study team, efforts to systematically follow these hyperlinks became time-consuming and confusing. In order to maximize the efficient use of online CEnR resources, clear guidelines for formatting access to materials on CTSA sites to be shared by multiple stakeholders are recommended to ensure minimal navigability issues for all potential stakeholders. Areas of website navigability have been previously identified, and include: “the clarity with which the target of a navigational element is described by that element (clarity of target), the clarity with which a navigational element conveys the underlying structure of site information (clarity of structure), and the degree to which the site content is appropriately subdivided or hierarchically organized with respect to the relationships between the content sections (logic of structure).” [50].

Finally, as our team reviewed resources, we found that the CEnR core competency domains we identified based on our review of CEnR competency literature did not adequately cover resource content concerning social determinants of health, cultural competency, and other aspects of community diversity essential to successful CEnR. The potential for CEnR to meaningfully account for the important roles of social determinants of health and cultural competency in healthcare research has long been recognized [51, 52]. The NCATS strategic plan emphasizes that translational research efforts should include an additional study of clinical implementation and social determinants of health [8]. Therefore, future CEnR resources need to include standardized nomenclature and training materials with a domain and associated competencies specific to social determinants of health and cultural competency.

Inconsistencies in the language used to define and describe various aspects and competencies related to CEnR was a limiting feature of resources included in this study. More than half of the resources had at least one domain scoring discrepancy between reviewers after the initial review, which suggested some challenges related to domain nomenclature. Due to the lack of standardized nomenclature to describe key CEnR concepts, it was challenging for reviewers to compare CEnR resources between CTSAs to ensure all similar resources were being captured in the study sample. This also created difficulty in identifying the CEnR core competency domains addressed within each resource, leading to many of the initial scoring discrepancies observed. Inconsistent language makes it difficult for stakeholders to search for relevant resources and difficult for novice CEnR stakeholders to understand key terminology and concepts. For example, we found that a variety of terms were used to refer to community partners (e.g., community stakeholders, patient partners, experience ambassadors, community advisors, etc.). Such a variety of terms and descriptions could be confusing to academic and community partners seeking to understand how community partners should be educated and participate CEnR projects. In a recent study, Eder and colleagues (2018) surveyed key informants at CTSAs in an effort to understand the consistency of CEnR definitions used among participating institutions. Their findings indicate there is a distinct need in CEnR for clearly delineated, specialized nomenclature in order to accurately describe and perform CEnR [53]. A standardized set of terminology and curricula of the core skills and resources are needed for CEnR to facilitate addressing gaps in CEnR and improving this body of knowledge [9]. Our findings also indicate that efforts to harmonize language in this area of research will have a high yield for the efficiency and effectiveness of future CEnR. As CTSAs attempt to leverage the use of existing resources and develop new ones, the standardization of CEnR language and concepts will be essential, and will align with the strategic plan of the NCATS to streamline the development of best practices, and facilitate more sharing and collaboration in translational science [8].

Current CEnR literature also does not describe how institutional factors, such as mentorship and resource availability to foster community outreach and engagement, can affect the development of community engagement. This is relevant to the creation of CEnR resources because universities that have a strong foundation of commitment to CEnR might emphasize competencies differently or develop curriculum differently than institutions with a weaker foundation [11]. The competencies needed for successful CEnR should describe specific attitudes, knowledge, and behaviors that a researcher must have to achieve both the goals of CEnR and of the partnership between the researcher and the community. Determining gaps in competency coverage of available resources can help address training opportunities for researchers and community partners prior to entering a partnership [54]. Much of the literature from our review recommends building a conceptual framework that identifies key competencies of CEnR that can be mapped to specific domains of knowledge [13]. To the co-authors’ knowledge, this is the first study to synthesize recognized components of CEnR competency into such defined domains. Identifying and defining core competency domains from the relevant literature will help in standardizing CEnR terminology for common use. There are additional efforts underway to identify core domains of CEnR competency using differing methodology, including a recent multi-institutional report from the Joint Working Group [6, 9, 11, 55]. Future CEnR research must synthesize such reports in a concerted effort to standardize CEnR competencies.

Some CTSAs provide a variety of outstanding CEnR education and training resources for community, academic, and clinical stakeholders. For example, the University of California San Francisco provided access to a variety of lectures and written materials intended for wide dissemination and use in education and training in CEnR [34]. The University of Illinois at Chicago also provided a variety of written training materials for CEnR, as well as several resources for working with specific vulnerable populations [36]. However, such quality was not uniform across resources, and there was great variation observed in the content, quality, and formats of the CEnR resources reviewed. Such variation presents stakeholders with difficulties in determining whether CEnR resources from CTSAs could be adapted for use in education and training in their potential CEnR projects. Identifying standard expectations for CEnR education and training resources to achieve would aid stakeholders in determining whether a resource could be utilized for their purposes. Therefore, there would be a benefit in developing grading criteria to help stakeholders assess the practical utility and adaptability of available CEnR resources. Future research should develop tools that help stakeholders evaluate the utility and adaptability of CEnR resources and should incorporate key aspects of CEnR resources observed in this study: content as it relates to core domains of CEnR competencies, intended audience, format, and accessibility.

The findings of this study should be interpreted while considering the following limitations. While the CEnR core competency domains used in this study were identified and defined using the expert consensus of the study team members, other CEnR experts may have identified and defined domains differently. Despite the interest in performing CEnR and the wealth of CEnR resources available from CTSAs, there is limited data and published literature related to the core competencies necessary for a successful scholarship of CEnR, as well as extreme variation in the methods and quality of literature concerning CEnR [6, 7]. Additional CEnR research is needed to establish universal CEnR core competency domains and their definitions. Since this study focused on the resources developed and provided by CTSAs, resources developed or provided by non-CTSA organizations that support CEnR education and training were not assessed. Finally, this study focused on the content of CEnR resources, and not the effectiveness of resources in providing adequate education and training. Future studies should examine the extent to which the intended audiences of these resources are able to successfully perform CEnR to fully assess the value of resources for use in CEnR education and training.

### Conclusion

This study suggests that CEnR resources are abundantly available online from CTSAs. Many available resources addressed at least one of the CEnR core competency domains identified by our study team. However, the lack of clear organizational structure within CTSA websites and standardized CEnR competency nomenclature render those resource platforms difficult to navigate, even for CEnR experts. Variability in the language used to describe essential components of CEnR is likely to be a major barrier to standardizing education and training for community stakeholders and researchers participating in CEnR. Future work should focus on consensus building toward a standardized nomenclature and guidelines to described CEnR competencies, and on incorporating competencies related to social determinants of health into CEnR education and training.

In order to build and sustain abilities to share resources for training and education in CEnR across CTSAs, these efforts are necessary. This study elucidated gaps in CEnR education and training that must be addressed. Gaps were identified not only in the content related to competencies in publicly accessible online CEnR resources, but also in the alignment of online resource access to ensure ready to access these invaluable resources generated by CTSAs. This study, combined with the knowledge generated through expert consensus panels, may be a platform on which to develop future resources, create standardized CEnR nomenclature, and bring forth the potential development of a comprehensive, systematically organized repository of tools specific to CEnR.
